# Establishing a Porcine Model of Small for Size Syndrome following Liver Resection

**DOI:** 10.1155/2017/5127178

**Published:** 2017-08-29

**Authors:** Mohammad Golriz, Maryam Ashrafi, Elias Khajeh, Ali Majlesara, Christa Flechtenmacher, Arianeb Mehrabi

**Affiliations:** ^1^Department of General, Visceral, and Transplantation Surgery, University of Heidelberg, Heidelberg, Germany; ^2^Department of General Pathology, University of Heidelberg, Heidelberg, Germany

## Abstract

**Background:**

Small for size syndrome (SFSS) is responsible for a high proportion of mortalities and morbidities following extended liver resection.

**Aim:**

The aim of this study was to establish a porcine model of SFSS.

**Methods:**

Twenty-four Landrace pigs underwent liver resection with a remnant liver volume of 50% (group A, *n* = 8), 25% (group B, *n* = 8), and 15% (group C, *n* = 8). After resection, the animals were followed up for 8 days and clinical, laboratory, and histopathological outcomes were evaluated.

**Results:**

The survival rate was significantly lower in group C compared with the other groups (*p* < 0.001). The international normalized ratio, bilirubin, aspartate transaminase, alanine transaminase, and alkaline phosphatase levels increased shortly after surgery in groups B and C, but no change was observed in group A (*p* < 0.05 for all analyses). The histopathological findings in group A were mainly mild mitoses, in group B severe mitoses and hepatocyte ballooning, moderate congestion, and hemorrhage, along with mild necrosis, and in group C extended tissue damage with severe necrosis, hemorrhage, and congestion.

**Conclusions:**

Combination of clinical, laboratory, and histopathological evaluations is needed to confirm the diagnosis of SFSS. 75% liver resection in porcine model results in SFSS. 85% liver resection causes irreversible liver failure.

## 1. Introduction

Improved surgical techniques and perioperative care have increased the use of extended hepatectomy (EH) for the treatment of primary and metastatic liver lesions [1, 2]. The main limiting factor for EH in many patients is an insufficient remnant liver volume (RLV) [3]. Insufficient RLV causes posthepatectomy liver failure (PHLF), which increases postoperative morbidity and mortality [4]. The term small for size syndrome (SFSS) was first used to describe clinical manifestations of refractory ascites, coagulopathy, encephalopathy, and hyperbilirubinemia following partial liver transplantation [5, 6] and now includes PHLF with insufficient RLV [7, 8].

Human clinical trials to investigate SFSS are impractical because of the high morbidity and mortality rates and ethical concerns. Therefore, it is necessary to establish experimental models that are similar to the human condition. The anatomy and physiology of pigs are similar to humans [9, 10]; therefore porcine models of EH are well-suited to studying the pathophysiology and prediction of SFSS [11–16]. However, SFSS has not been prospectively evaluated following EH with regard to the clinical, laboratory, and histopathological findings. The aim of this study was to establish a porcine model of SFSS by analyzing the RLV following different degrees of liver resection, together with the clinical, laboratory, and histopathological outcomes.

## 2. Material and Methods

### 2.1. Study Design

**Figure 1:**
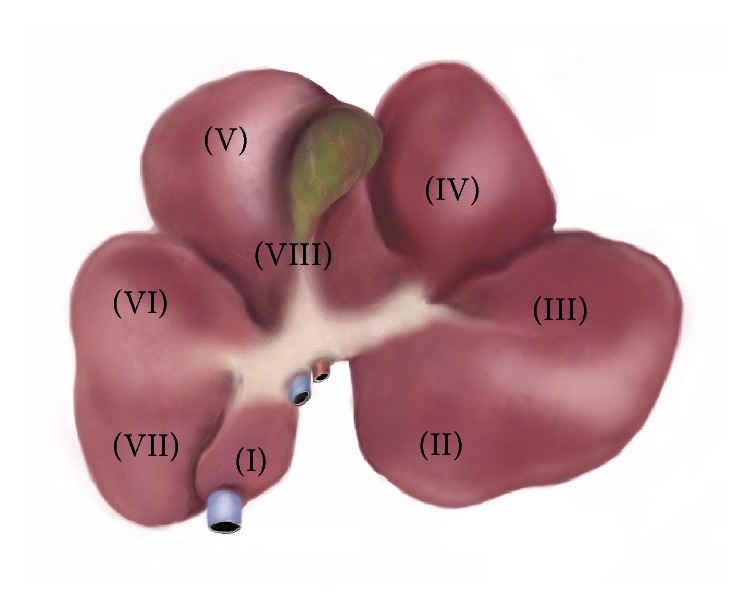
Segmental anatomy of the porcine liver. Caudate lobe: segment (I). Left lateral lobe: segments (II) and (III). Left medial lobe: segment (IV). Right medial lobe: segments (V) and (VIII). Right lateral lobe: segments (VI) and (VII).

Twenty-four female Landrace pigs with a body weight of 26 kg to 35 kg were used in this study. Animals were reared in the interdisciplinary biomedical research center (IBF) of the University of Heidelberg. The animals were weighed and blood samples were taken prior to surgery. Animals were allocated to one of three groups, with eight pigs in each group. In group A, 50% of the liver was resected (segments (II), (III), and (IV)). In group B, 75% of the liver was resected (segments (II), (III), (IV), (V), and (VIII)), and in group C, 85% of the liver was resected (segments (II), (III), (IV), (V), (VI), and (VIII)) (Figure 1). After resection, the pigs were observed for 8 days and clinical, laboratory, and histopathological outcomes were evaluated.

### 2.2. Anesthesia and Surgical Procedure

After 12 hours of fasting with free access to water, all animals underwent general anesthesia using our standard protocol [11]. Following a midline incision laparotomy, the liver was mobilized and the hepatoduodenal ligament was prepared. Liver resections were performed according to our standard method [17] using stapler (Endo GIA™ Universal Stapler, Covidien, Minneapolis, USA). After resection, electrocoagulation and hand-suturing with Polybutester 3-0 (Novafil™, Covidien, Minneapolis, USA) were performed to achieve complete hemostasis.

### 2.3. Postoperative Care and Assessment

#### 2.3.1. Medications

Postoperative medication included daily intravenous (IV) administration of 3 mg buprenorphine hydrochloride (Temgesic, Reckitt Benckiser, Switzerland) and daily IV administration of 500 mg metamizole sodium (Novalgin, Sanofi-Aventis, Germany) for pain relief, prophylactic antibiotic therapy with twice daily administration of 250 mg metronidazole (Flagyl®, Sanofi-Aventis, Germany) and 375 mg sultamicillin (Unacid®, Pfizer Pharma GmbH, Berlin, Germany), and daily administration of 40 mg pantoprazole (HEXAL AG, Holzkirchen, Germany).

#### 2.3.2. Laboratory Assessments

During the 8-day follow-up, the clinical and laboratory status of the animals were continuously assessed. Histopathological analysis was performed when the animals were sacrificed. The pigs were monitored twice daily and the general conditions, behavior, abdominal distention, food intake, and defecation were observed. Laboratory evaluation was performed before resection (*T*0), one hour after resection (*T*1), one day after resection (*T*2), and on the day of sacrifice (*T*3). Laboratory assessments included measurements of total bilirubin, aspartate transaminase (AST), alanine transaminase (ALT), alkaline phosphatase (ALP), and albumin levels and calculation of the international normalized ratio (INR).

#### 2.3.3. Histopathological Evaluations

To evaluate histopathological changes after resection, an open wedge liver biopsy was obtained from the RLV when the animals were sacrificed. Samples were fixed in 10% formaldehyde, embedded in paraffin, and sectioned for hematoxylin and eosin (H&E) staining. The sections were reviewed by a pathologist who was blinded to the extent of liver resection. The histopathological samples were assessed for degree of mitoses, inflammation, periportal/septal edema, hepatocyte ballooning, congestion, hemorrhage, and necrosis in line with previously published studies [18–21]. Samples were graded using a severity scale ranging from 0 to +++, with 0 indicating no pathological changes, + indicating mild changes, ++ indicating moderate changes, and +++ indicating severe/significant changes. The results with significant mitosis, moderate to severe inflammation, edema, and hepatocyte ballooning were defined on behalf of SFSS. Massive necrosis and hemorrhage without mitoses and regeneration was defined as irreversible PHLF [18, 20, 21].

### 2.4. Statistical Analysis

Statistical analysis was performed using statistical package for social sciences (SPSS), version 23 (IBM Corp. Released 2015. IBM SPSS Statistics for Windows. Armonk, NY: IBM Corp). Continuous variables are expressed as mean ± standard deviation (SD). To analyze survival rates, the Kaplan-Meier method was applied and mean survival rates were compared using the log-rank test. Mean baseline and outcome values were compared at each time point using one-way analysis of variance (ANOVA). A repeated measures ANOVA model was used to compare the overall differences among laboratory findings between three study groups. In all tests, a *p* value of less than 0.05 was considered statistically significant.

### 2.5. Animal Rights

Animal care conformed to institutional guidelines of the Animal Care Facility at the University of Heidelberg. Animals were sacrificed under deep anesthesia at the end of the study protocol or when ethically indicated, with intravenous injection of potassium chloride (2 mmol/kg). The study protocol was approved by the German Committee for Animal Care, Karlsruhe, Germany (AZ: 35-9185.81/G-45/12).

## 3. Results

Twenty-four Landrace pigs with a mean body weight of 30.2 ± 2.9 kg were divided into three groups before undergoing standard liver resection surgery: group A (*n* = 8) with 50% RLV, group B (*n* = 8) with 25% RLV, and group C (*n* = 8) with 15% RLV. There were no significant differences in body weight and baseline laboratory data between the three groups (Table 1).

### 3.1. Laboratory Data

**Figure 2:**
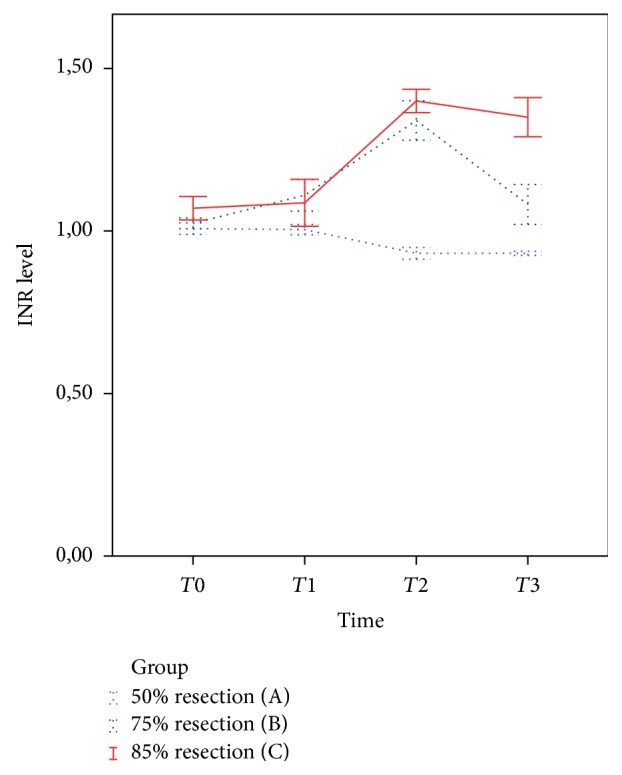
International normalized ratio (INR) levels at baseline and after liver resection. *T*0 (baseline, *p* = 0.199); *T*1 (one hour after surgery, *p* = 0.148); *T*2 (one day after surgery, *p* < 0.001); *T*3 (at sacrifice, *p* < 0.001). INR levels were significantly lower in group A compared with groups B and C (*p* < 0.001) at different time points. Error bars show standard error of the mean.

Prior to and one hour after the operation, there were no significant differences in the INR between the three groups (Figure 2, *p* = 0.20 and 0.15, resp.). One day after liver resection and on the day of sacrifice, INR values were significantly lower in group A compared with groups B and C (Figure 2, *p* < 0.001). The INR reached its peak level on the first postoperative day in groups B and C. INR values decreased to near preoperative values on the day of sacrifice in group B. However, the INR did not change in group C. As depicted in Figure 2, a repeated measures ANOVA revealed that the INR was significantly lower in group A compared with groups B and C over the follow-up period (*p* < 0.001).

**Figure 3:**
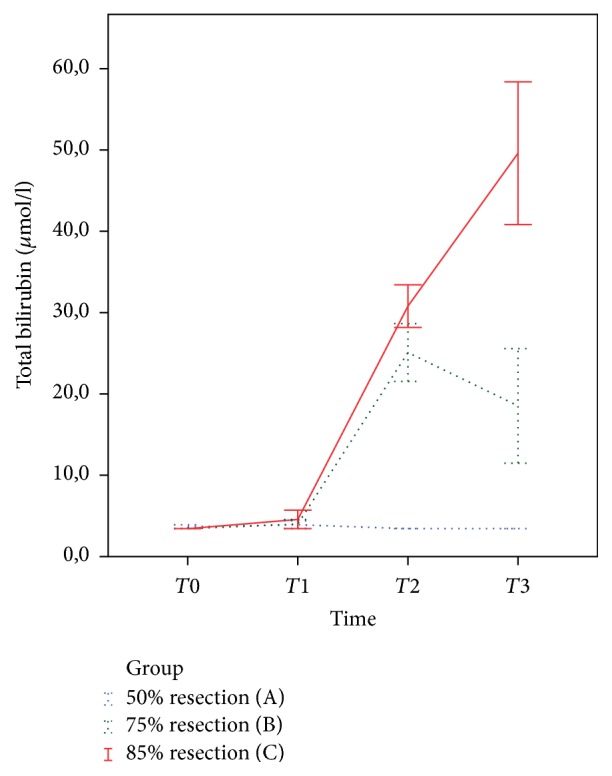
Total bilirubin levels at baseline and after liver resection. *T*0 (baseline, *p* = 0.559); *T*1 (one hour after surgery, *p* = 0.804); *T*2 (one day after surgery, *p* < 0.001); *T*3 (at sacrifice, *p* < 0.001). Total bilirubin levels were significantly lower in group A compared with groups B and C (*p* < 0.001) at different time points. Error bars show standard error of the mean.

Mean total bilirubin levels at baseline and one hour after liver resection were not significantly different between the three groups (Figure 3, *p* = 0.559 and *p* = 0.804, resp.), but total bilirubin levels were significantly lower in group A on the first postoperative day (*p* < 0.001) and on the day of sacrifice (*p* < 0.001). The total bilirubin levels increased in groups B and C, but started to decrease slightly in group B and continued to increase in group C (Figure 3). Repeated measures ANOVA revealed that total bilirubin was significantly lower in group A during follow-up compared with the other groups (*p* < 0.001).

AST, ALT, ALP, and albumin levels for all study groups at each time point are shown in Table 2. The mean levels of AST, ALT, and ALP did not differ significantly between the three groups at baseline and one hour after liver resection but were significantly higher in groups B and C one day after resection and on the day of sacrifice. Albumin levels were significantly lower in groups B and C one hour after resection and on the day of sacrifice than group A. Repeated measures ANOVA revealed significant differences in AST, ALT, ALP, and albumin changes between the groups during the follow-up period.

### 3.2. Survival

**Figure 4:**
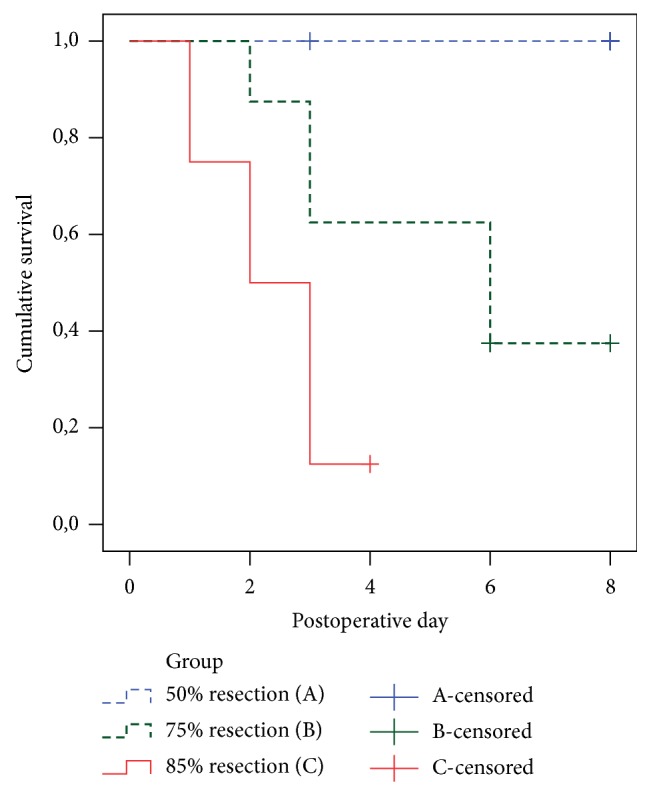
Kaplan-Meier survival graphs of the study groups. Survival rates were significantly different in three study groups (log-rank test *p* < 0.001). Censored cases died due to unknown causes or were alive at the end of the follow-up time.

The animals were followed up for 8 days after liver resection and then sacrificed. According to the Animal Care Facility protocols, the follow-up was ended and animals were sacrificed if the clinical situation exacerbated with severe signs of acute liver failure. Seven out of eight pigs in group A survived to the end of the study period with recovered liver function; one was sacrificed on the third postoperative day due to severe ileus and abdominal distention and inability to take food. In group B, only one animal survived for 8 days; one died on the second postoperative day, and the other six survived at least 3 days (range: 3 to 6 days). In group C, seven animals died within the first 3 postoperative days, and one died on the fourth postoperative day. The log-rank test revealed that the survival rate in group C was significantly lower than the other groups (*p* < 0.001). Kaplan-Meier survival curves for each group are shown in Figure 4.

### 3.3. Pathologic Findings

**Figure 5:**
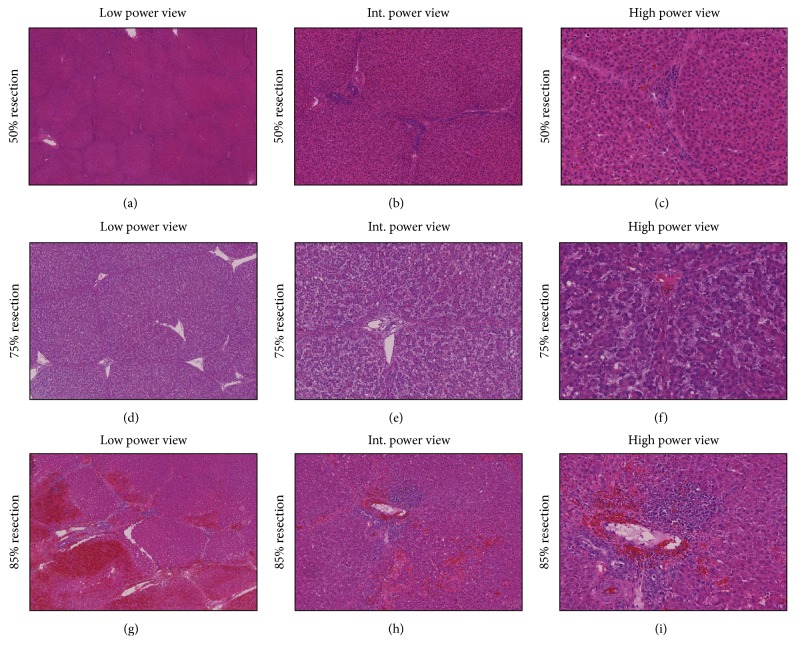
Composite of liver biopsies of 50%, 75%, and 85% liver resection at the time of sacrifice with low (a, d, and g), intermediate (b, e, and h), and high power view (c, f, and i). The low power view shows no significant change in 50% hepatectomy group. Normal hepatocytes and unchanged hepatic structures are seen (a). In the high power views only few mitoses are seen ((b) and (c)). The low power view of liver after 75% liver resection is shown in (d). Portal and periportal edema and significant elevated mitoses are prominent findings in 75% hepatectomy group ((e) and (f)). The low power view of 85% resection reveals extensive hemorrhage (g). Necrosis, bleeding, and inflammation in periportal area are shown in high power view (h and i). Int.: intermediate.

Table 3 shows the detailed histopathological characteristics of each study group. Examination of H&E stained sections by light microscopy revealed mainly mild (+) mitoses in group A (50% RLV) (Figures 5(a), 5(b), and 5(c)). In group B (25% RLV), severe (+++) hepatocyte ballooning, moderate (++) congestion, hemorrhage, inflammation, periportal/septal edema, and mild (+) necrosis occurred (Figures 5(d), 5(e), and 5(f)). Group B livers showed significant (+++) mitoses at the time of sacrifice, but only mild (+) mitoses were seen in group C (75% RLV). Moreover, in group C, severe (+++) necrosis, hemorrhage, and congestion were observed, which reflected extensive tissue damage (Figures 5(g), 5(h), and 5(i)). Other findings in the group C were mild (+) inflammation, periportal/septal edema, and hepatocyte ballooning.

## 4. Discussion

Advances in the field of hepatobiliary surgery have led to an increase in curative extended liver resection [1, 2]. However, SFSS remains a challenging issue and the size of the remnant liver is a major limiting factor of curative resection [3]. To investigate this problem, several studies have tried to develop an optimal experimental model for SFSS. [5, 12, 18, 22–25]. Even, Mohkam et al. published recently a review of literature in this regard [26]. However, the majority of published studies have focused on SFSS following partial liver transplantation and not liver resection. Also, the few studies that used a large animal model with focus of SFSS following extended liver resection [23, 25, 27, 28] did not consider the clinical evaluations, laboratory findings, and histopathological results together.

For example, in the study of Xia et al. [23] the surgical methods, survival rates, and laboratory outcomes were well described and the authors concluded that left EH plus VI segmentectomy provides a simple SFSS model. However, no differences in laboratory findings and portal pressure gradient between the left EH group and left EH plus VI segmentectomy group were found and the diagnoses of SFSS and irreversible PHLF were not confirmed by pathologic evaluation. Furthermore, the establishment of SFSS model in this previous study was based on a lower survival rate in left EH plus VI segmentectomy group compared with left EH group. In the study of Mohkam et al. [28] there is no follow-up data of the pigs after 70% and 90% resection but the authors believe that 90% hepatectomy is a reliable model for studying SFSS. An upper limit of the portal vein flow > 250 ml/min/100 g for SFSS following liver transplantation in clinical setting has been suggested by Troisi et al. [29, 30]. However, in the study of Mohkam et al. [28] the portal vein flow levels following 75% resection exceeded 250 ml/min/100 g level and it is quite likely that pigs would have developed SFSS syndrome if the follow-up continued [31]. In 2004, Court et al. [27] introduced the first porcine model of SFSS for the study of liver regeneration. They concluded that both models of 80% (trilobectomy) and 90% (subtotal) hepatectomy allow easy assessment of posthepatectomy liver function and regeneration. However, the 90% resection group with higher death rate represents a model of critical residual liver parenchyma. In a recent review of the porcine models for the study of SFSS, Mohkam et al. suggested that resection of all segments of the liver except segment 1 (subtotal hepatectomy) is the best model of SFSS after hepatectomy [26].

The most important issue in this regard that may be confusing and affect the interpretations about the volume of the resection to achieve SFSS can be explained as follows:

1. Only based on phrasal definition, every small for size remnant liver following EH can be considered as SFSS; however, it has to be distinguished between the proper model of SFSS and the irreversible acute PHLF.
2. It is true that a 10% RLV following EH is small for size; however, this RLV will be damaged rapidly and irreversibly following the resection.
3. Again, only based on definition every modulation that prepares the RLV a window period for regeneration would be effective in preventing SFSS; however, in the clinical setting this window period should be tolerable and not lead to death until the liver regenerate itself. For example, following 90% liver resection the RLV will be rapidly damaged through the high portal vein flow and there is no chance for the liver to regenerate itself. Even a modulation of the transhepatic flow, which could potentially give the liver a window period for regeneration, cannot be tolerated and the animal or patient dies.
4. Since the aim of establishment of an animal model of SFSS is a better understanding of this clinical syndrome as well as studying the possible preventive and curative methods, we strongly believe that, in a porcine model with normal parenchyma, 90% liver resection cannot be considered as SFSS model. Following 90% liver resection there is a very low chance for the liver to be rescued through any modulation.
5. An optimal model for SFSS can be achieved following a major resection when the situation is still reversible and can be prevented or managed. In the clinical setting, it is also not logical to resect 90% of a liver of a patient, because there is no survival chance even through hepatic inflow modulation.

In the present study, based on the experiences of Troisi et al. [29, 32] in small for size grafts in living donor liver transplantation, we established a porcine model of SFSS with consideration of the histopathologic, laboratory, and clinical findings. Histopathologic changes in 75% liver resection in our study support the establishment of SFSS in this group. We selected a porcine model because the pig liver has a similar size and anatomy to humans and therefore provides a greater scope for surgical procedures than small animal models [33, 34]. Furthermore, the segmented nature of the porcine liver makes anatomical liver resection easy to perform, similar to the human situation [9]. Since the vena cava is enclosed by the liver parenchyma in pigs, therefore it is easier and safer to perform left EH than right EH in porcine models. To compare our results with baseline data, we defined a control group and resected 50% of the liver in this group. The only histopathologic finding after 50% liver resection was mild mitoses. The laboratory findings were normal in the control group during follow-up and the survival rate was significantly higher than the other two groups. In this group only one pig was sacrificed during the follow-up due to severe ileus. However, the pathologic findings of the liver did not show any signs of liver damage. After 75% liver resection, the pigs showed histopathological signs of SFSS, including mitoses, severe hepatocyte ballooning, moderate inflammation, edema, and congestion as previously showed through Demetris et al. [20, 29]. Elevated INR, bilirubin, AST, ALT, ALP, and decreased albumin and also postoperative observation of the animals again indicated SFSS in the group with 75% resection. In this group 5 pigs survived more than 3 days and one of them survived for complete 8-day follow-up. Among 7 pigs which died, cause of death in 5 was liver failure. These correlate with the results of Dahm et al. [5, 20, 35]. Although the mean survival rate was lower in our 75% resection group compared with previous findings of Xia et al. [23], this can be explained by our ethical policy, which did not let further follow-up of animals with severe signs and complications of SFSS.

Following 85% liver resection, the liver failed to regenerate. Histopathological evaluation revealed massive necrosis, hemorrhage, congestion, and low mitoses in all animals in group C, indicating irreversible PHLF [36]. Additional indications of irreversible PHLF were a rapid rise in INR, elevated bilirubin, impaired liver function, and a rapid deterioration of the general condition as a sign of acute irreversible PHLF [35, 36]. Only one animal survived more than 3 days in this group. The cause of death in all except one of the pigs was liver failure. Severe necrosis and microhemorrhage caused irreversible organ failure leading to early death, indicating that 15% RLV does not represent a suitable model for investigating SFSS. These findings are in agreement with previous reports that 85% liver resection causes fatal hepatic failure [14, 37, 38]. The main difference between groups B and C is the reversibility of the changes in the laboratory and pathological parameters after hepatectomy. The pathological findings revealed high rate of mitoses and regeneration in group B versus massive necrosis and only minor mitoses in group C. Thus, immediately after 85% liver resection the remnant liver starts an irreversible damage, leading to an inevitable and lethal liver failure. But after 75% liver resection some of the animals can survive only for few days (more than 3 but less than 7 days) and some others are able to overcome the acute postoperative changes while the liver regenerates itself sufficiently. Therefore, 75% liver resection is a proper model for SFSS, because the pigs have a chance to survive, and preventive and therapeutic interventions can be studied on them. We observed no differences in the laboratory results after 75% and 85% liver resection, demonstrating that laboratory findings cannot solely distinguish between SFSS and irreversible PHLF in the first postoperative days. In the case of elevated INR and bilirubin, animals should be closely observed for signs of general deterioration. In the present study, we established a porcine model of SFSS according to the RLV, clinical, laboratory, and histopathological findings. Since the transhepatic flow plays an important role in the pathophysiology of SFSS [29, 39], a limitation of our study was that we did not measure the transhepatic flow in this study. We suggest that this should be considered in all SFSS studies.

## 5. Conclusions

In conclusion, porcine model is a feasible and appropriate model for studying the pathophysiology, prediction, prevention, diagnosis, and management of SFSS. Resection of segments (II), (III), (IV), (V), and (VIII) of the porcine liver leaves a 25% RLV. This causes known symptoms of SFSS and can be used for experimental studies. Finally, to have an appropriate experimental model of SFSS, we suggest a comprehensive assessment of the combination of clinical, laboratory, and histopathologic findings.

## Figures and Tables

**Table 1:** Body weight and laboratory results before liver resection.

	Group A (mean ± SD)	Group B (mean ± SD)	Group C (mean ± SD)	*p*
Body weight (kg)	30.5 ± 3.4	30.4 ± 3.3	30.0 ± 2.6	0.938
INR	1.00 ± 0.05	1.02 ± 0.04	1.06 ± 0.05	0.061
Albumin (g/l)	32.1 ± 3.3	30.4 ± 2.9	32.3 ± 3.2	0.420
Total bilirubin (*μ*mol/l)	3.76 ± 0.86	3.42 ± 0.00	3.42 ± 0.00	0.122
AST (U/l)	46.5 ± 10.1	60.6 ± 37.6	71.6 ± 65.6	0.530
ALT (U/l)	43.6 ± 6.6	50.5 ± 10.6	53.9 ± 9.4	0.092
ALP (U/l)	170.9 ± 30.8	149.4 ± 42.0	144.4 ± 42.8	0.369

A: 50% resection; B: 75% resection; C: 85% resection; SD: standard deviation; INR: international normalized ratio; AST: aspartate transaminase; ALT: alanine transaminase; ALP: alkaline phosphatase.

**Table 2:** Laboratory findings at baseline and during the follow-up period.

	*T*0	*T*1	*T*2	*T*3	*p* ^*∗*^
Albumin (mean [95% CI])					

Group A	31.5 (28.8–34.1)	29.5 (26.7–32.2)	31.2 (28.3–34.1)	33.3 (31.7–34.8)	0.016
Group B	30.7 (27.1–34.2)	23.4 (19.3–27.4)	30.2 (29.0–31.5)	27.6 (24.1–31.2)	
Group C	30.1 (26.3–34.0)	28.7 (16.2–41.2)	30.7 (19.4–42.0)	26.2 (16.1–36.2)	
*p* ^*∗∗*^	0.783	0.028	0.826	0.003	

AST (mean [95% CI])					

Group A	46.6 (36.5–56.7)	62.9 (50.3–75.4)	59.7 (−1.9–121.4)	29.9 (25.1–34.6)	0.001
Group B	49.0 (41.0–57.0)	249.0 (16.8–481.2)	811.2 (493.3–1129.0)	276.5 (45.4–507.6)	
Group C	49.3 (37.9–60.8)	127.7 (−86.1–341.5)	502.7 (345.4–659.9)	541.3 (−610.3–1693.0)	
*p* ^*∗∗*^	0.845	0.096	<0.001	0.017	

ALT (mean [95% CI])					

Group A	41.2 (33.6–48.7)	39.0 (34.6–43.4)	54.0 (42.4–65.5)	37.2 (28.1–46.3)	0.003
Group B	51.0 (39.7–62.3)	64.2 (25.7–102.6)	110.3 (79.8–140.9)	81.0 (43.7–118.3)	
Group C	54.3 (35.4–73.3)	50.7 (11.8–89.5)	83.7 (51.2–116.1)	115.3 (21.2–209.5)	
*p* ^*∗∗*^	0.116	0.307	0.004	0.010	

ALP (mean [95% CI])					

Group A	170.7 (140.0–201.4)	168.0 (142.3–193.7)	148.1 (135.5–160.0)	101.9 (89.6–114.1)	0.001
Group B	144.6 (85.2–204.0)	157.2 (96.5–217.9)	468.6 (341.2–596.0)	317.0 (72.5–561.5)	
Group C	157.3 (121.4–193.3)	215.3 (−64.3–495.0)	435.0 (270.7–599.2)	523.0 (−405.6–1451.6)	
*p* ^*∗∗*^	0.497	0.386	<0.001	0.020	

^*∗*^ *p* values of repeated measures ANOVA; ^*∗∗*^*p* values of one-way ANOVA; A: 50% resection; B: 75% resection; C: 85% resection; CI: confidence interval; *T*0: baseline; *T*1: one hour after resection; *T*2: one day after resection; *T*3: at sacrifice; INR: international normalized ratio; AST: aspartate transaminase; ALT: alanine transaminase; ALP: alkaline phosphatase.

**Table 3:** Scored pathological features.

	Mitoses	Necrosis	Congestion/hemorrhage	Inflammation	Periportal/septal edema	Hepatocyte ballooning
Group A	+	0	0	0	0	0
Group B	+++	+	++	++	++	+++
Group C	+	+++	+++	+	+	+

0: no significant changes; +: mild changes; ++: moderate changes; +++: significant (severe) changes; A: 50% resection; B: 75% resection; C: 85% resection.
